# Fine-root and leaf acquisitive traits decoupled from chloride accumulation in reflecting the differential salinity tolerance among *Prunus* hybrids

**DOI:** 10.3389/fpls.2024.1502201

**Published:** 2025-01-08

**Authors:** Shuangxi Zhou, Rob R. Walker, Everard J. Edwards

**Affiliations:** CSIRO, Glen Osmond, Adelaide, SA, Australia

**Keywords:** acquisition-conservation trade-off, almond rootstock, ion accumulation, root distribution, root morphology, salt stress, soil environment change, stress acclimation

## Abstract

Improving crop salinity management requires enhanced understanding of salinity responses of leaf and fine-root traits governing resource acquisition, ideally in relation to ion accumulation at intra- or inter-specific levels. We hypothesized that these responses are coupled towards integrated resource conservation for plants under prolonged salt treatment. We tested the hypothesis with a glasshouse experiment on saplings of six contrasting *Prunus* hybrids, subjected to either control or salt treatment (reverse osmosis water versus 3.3 dS m^-1^ chloride solution containing mixed cations). Sample collections were carried out at 30 and at 60 days after the start of treatments. All six hybrids showed significantly higher lamina chloride concentration in response to salt treatment, with GF677 accumulating a lower concentration than the other five hybrids. There was significantly lower specific leaf area (SLA) in ‘Monegro’ and lower root tissue density (RTD) in ‘Nemaguard’ after 60 days – but not 30 days – of salt treatment. No hybrid showed concurrent significant decrease of SLA and specific root surface area (SRA) under salt treatment. The *a priori* known salinity-sensitive hybrid ‘Nemaguard’ not only showed decreased RTD and a negative relationship between root biomass and salt treatment duration, but also showed increased SRA without notable change of average root diameter. Lamina chloride accumulation and leaf gas exchange response were closely correlated along a gradient towards resource conservation from control to salt-treated plants in all hybrids, which was orthogonal to another gradient characterized by a hybrid-dependent modification of SLA, SRA, RTD and percentage of root length within the finest diameter class. This study highlighted the intraspecific differential resource investment strategies, reflected by the hybrid-specific salinity-response coordination among leaf and fine-root acquisitive traits.

## Introduction

Globally, as tree crop production expands and/or drought conditions occur with limitations to availability of good quality water for irrigation, growers are increasingly considering the use of saline water for irrigation. Salinity is one of the most severe abiotic factors imposing detrimental impacts on plant growth and development worldwide, especially in arid and semi-arid areas (Munns and Tester, 2008; Galvan-Ampudia and Testerink, 2011).

Different plant types under salt stress can accumulate sodium (Na^+^) and chloride (Cl^–^) to different extents (Maas, 1986; Läuchli et al., 2008). Most fruit crops, including almonds, are considered to be salt-sensitive (Maas, 1986; Ottman and Byrne, 1988), and particularly sensitive to the predominant anion – Cl^–^ – in many saline soils (Bernstein, 1980; Lupo et al., 2022). High Cl^–^ concentration can be toxic to plants by causing membrane damage, enzyme inhibition which affects photosynthetic processes (Tavakkoli et al., 2010), degradation and reduction of leaf chlorophyll (James et al., 2002; Tavakkoli et al., 2010), and inhibition of photosynthetic capacity (Seemann and Critchley, 1985). In addition to the accumulation of Na^+^ and/or Cl^–^ during salt treatment, the accumulation of other mineral elements may also be affected, which can lead to an imbalance of essential nutrients (Ruiz et al., 1997; Lupo et al., 2022; Shelden and Munns, 2023).

The degree of salinity tolerance of plants in general is thought to be related to their capacity to exclude salt from the shoot and/or their capacity to tolerate high concentrations of accumulated ions such as Na^+^ and Cl^–^ in tissues (Munns and Tester, 2008; Lupo et al., 2022). However, evidence on the correlation between salinity tolerance and tissue ionic concentrations has been mixed, as there are reports on inverse correlation (e.g., Flowers and Yeo, 1981), no correlation (Genc et al., 2007) or genotype-dependent relationships (e.g., Greenway and Munns, 1980; James et al., 2002), indicating that Na^+^ and/or Cl^–^ exclusion from shoot tissues is not always correlated with salinity tolerance in plants. For some crop types, e.g., grapevine, under field conditions, there is a relationship between rootstock capacity for salt exclusion and overall salt tolerance as measured by scion vigor and yield (Walker et al., 2002). *Prunus* rootstocks differ significantly in their capacity to exclude both Cl^–^ and Na^+^ ions during salt treatment. In a study involving 14 *Prunus* rootstocks, the majority accumulated significantly more Cl^–^ than Na^+^, ranging from around 2-fold higher for ‘Hansen 536’ to slightly higher for ‘Bright’s Hybrid’ (Sandhu et al., 2020). High Cl^–^ concentration is often observed in tissues of plants adapted to saline soil (Kingsbury and Epstein, 1986; Gorham, 1990; Lupo et al., 2022; Shelden and Munns, 2023).

Salinity-induced plant adaptation responses can lead to modification of resource-acquisitive traits of foliar tissue such as specific leaf area (SLA) (e.g. Romero-Aranda et al., 2001; Uchiya et al., 2016) and root tissue such as specific root surface area (SRA), root tissue density (RTD), average root diameter and root diameter distribution (e.g., Lovelli et al., 2012; Lupo et al., 2022; Shelden and Munns, 2023), that may contribute to the plant’s capacity to cope with salt stress (Julkowska and Testerink, 2015). SLA, characterizing the light acquisitive capacity, and SRA, characterizing root absorptive capacity, are key traits associated with plant resource acquisition and investment strategies (Cheng et al., 2016; Zhou et al., 2020; Lupo et al., 2022). RTD can reflect plant investment trade-off between building resource-expensive but resilient roots or cheap but fragile and less-resilient roots (Craine et al., 2001; Lupo et al., 2022; Zhao et al., 2024). Root diameter can reflect root hydraulics and lifespan (Kong et al., 2019; Zhao et al., 2024). Root diameter distribution – the distribution of root length according to root diameter classes, particularly the percentage of root length within the finest diameter class, can also reflect the fineness of the root system (Zhou et al., 2020). Enhanced SRA, decreased RTD and average root diameter have been linked to improved salt exclusion of grapevine rootstocks through reduced salt uptake from the soil (Lupo et al., 2022). Partly due to the logistical difficulties in measuring root traits, studies concurrently exploring the salinity responses of fine-root and leaf acquisitive traits are very limited (Shelden and Munns, 2023).

The discrepancy among studies on the correlation between plant salinity tolerance and Na^+^ and/or Cl^–^ accumulation raises the necessity to test (1) the responses of leaf and fine-root traits governing plant resource acquisition and investment under stress (Eissenstat et al., 2000; Zhou et al., 2020), and (2) their response covariation at inter- and/or intra-specific levels besides ion accumulation (Julkowska et al., 2014; Uchiya et al., 2016; Lupo et al., 2022; Shelden and Munns, 2023). Meanwhile, salinity damage in tree crops is also largely associated with rootstock type and the duration of plant exposure to the saline water (Company and Gradziel, 2017; Lupo et al., 2022). The salinity-induced adaptation responses above- and/or below-ground, such as morphological modification in leaf and/or root acquisitive traits, are not likely to develop during short-term salinity experiments (Romero-Aranda et al., 2001; Julkowska and Testerink, 2015). There is a lack of knowledge on the inter- and/or intra-specific variation in the degree of leaf and root trait modifications during prolonged salt treatment (Julkowska et al., 2014). Salinity experiments that standardize factors including plant growth stage, soil texture, soil water and nutrient status, the specific ions contributing to salinity in the root zone, plant traits and genetic background are much needed (Rengasamy, 2010; Butcher et al., 2016; Uchiya et al., 2016; Lupo et al., 2022; Shelden and Munns, 2023).

We hypothesized that responses of leaf and fine-root acquisitive traits are coupled towards integrated plant resource conservation for plants under prolonged salt treatment. We tested the hypothesis using a glasshouse experiment on saplings of *Prunus* hybrids with *a priori* known contrasting salinity sensitivity. Seedling plants grown in sandy loam soil were imposed with either control (reverse osmosis water) or salt treatment (3.3 dS m^-1^ Cl^–^ solution with mixed cations) for 30 and 60 days, respectively. We measured the key variables associated with the hypothesis (i.e., leaf and fine-root acquisitive traits, leaf gas exchange, and concentration of the predominant ion, Cl^–^, in lamina), and investigated their interrelationships, in particular: (1) whether the modification of above- and/or below-ground traits critical to plant resource economy would be different under prolonged salt treatment (i.e., 60 versus 30 days), (2) besides lamina Cl^–^ accumulation, whether salt treatment would lead to analogous modifications between above- and below-ground pairs of acquisitive traits (i.e., SLA and SRA), and (3) whether the degree of trait modifications, if any, would differ among congeneric hybrids – reflecting intraspecific differential salinity tolerance. Our goal was to test the hypothesis and thereby contribute to a better understanding of the interrelationships among salinity tolerance, lamina Cl^–^ concentration, and leaf and fine-root acquisitive traits – at the intraspecific level.

## Materials and methods

### Choice of hybrids

Rootstock can affect water and nutrient uptake and limit the uptake and transport of salt (Lupo et al., 2022). The six rootstock hybrids – ‘Bright’s Hybrid’, ‘Felinum’, ‘GF677’, ‘Monegro’, ‘Nemaguard’, and ‘Viking’ – are commercially utilized by growers in California and Australia – the top two almond planting regions of the world, where salinity is a potential industry challenge. For almond growers in Australia, large production areas (e.g., the Murray–Darling Basin) use low salinity water for irrigation, i.e., that extracted from the Murray River having an electrical conductivity (EC) of 0.3 to 0.4 dS m^-1^. Irrigation water electrical conductivities can, however, increase during drought, with potential for EC of the soil solution to increase further when insufficient water is available to leach salts that may have accumulated as water evaporates from the soil surface (Lanyon, 2011). Peach rootstocks (e.g., ‘Nemaguard’) are usually more salinity-sensitive than almond × peach hybrid rootstocks (e.g., ‘Bright’s Hybrid’, ‘Felinem’, ‘GF677’ and ‘Monegro’) and complex hybrids (e.g., ‘Viking’) (Company and Gradziel, 2017). ‘Felinem’ and ‘Monegro’ were primarily bred for root-knot nematode resistance, and experiments screening their salinity sensitivity are rare. ‘Nemaguard’ has been reported as very salinity-sensitive (El-Motaium et al., 1994; Company and Gradziel, 2017). ‘Bright’s Hybrid’ (El-Motaium et al., 1994; Sandhu et al., 2020), ‘GF677’ (Najafian et al., 2008; Ouraei et al., 2009; Dejampour et al., 2012) and ‘Viking’ (Company and Gradziel, 2017; Sandhu et al., 2020) have been described as more salinity-tolerant.

### Plant material, growth conditions, and experimental design

In February 2015, one-year-old ungrafted saplings of six *Prunus* hybrids were transplanted into 2.4-litre pots with 2 liters of sandy loam soil which was evenly mixed with slow-release fertilizer. The plants were grown in a glasshouse transparent to sunlight under a 27°C/20°C diurnal temperature cycle and maintained in a moist condition for three months to allow establishment. Two night-break lights were used to extend the daily light length for three more hours to prevent short-day responses as the season was entering into autumn. For each hybrid, plants of similar sizes were randomly assigned to one of the two watering treatments – reverse osmosis water as control treatment or 3.3 dS m^-1^ Cl^–^ solution with mixed cations as salt treatment – in the glasshouse during May – July 2015. The Cl^–^ solution was comprised of 20 mmol NaCl, 3.3 mmol MgCl_2_ and 3.3 mmol CaCl_2_ – with a total of 33.2 mmol Cl^–^ per liter and the EC of the Cl^–^ solution measured as 3.3 dS m^-1^. The salt treatment in this experiment used the solution with an EC of 3.3 dS m^-1^ because saline irrigation water with EC above 3 dS m^-1^ has been reported to adversely affect chlorophyll content and fluorescence parameters in almond leaves (Ranjbarfordoei et al., 2006). The salinity-sensitive ‘Nemaguard’ has been reported to show a decline of almond yield at the salinity level of 2.5 dS m^-1^ (Company and Gradziel, 2017). Every day, each pot was irrigated with 500 mL reverse osmosis water or Cl^–^solution (more than the field capacity), and the drainage of each pot was checked to avoid waterlogging or salt accumulation in soil.

Plants were harvested at three time points – the day before the beginning of treatments, then at 30 days and 60 days of treatments, respectively. Six plants of ‘Bright’s Hybrid’, ‘Felinum’, ‘Monegro’, and ‘Nemaguard’, and five plants of ‘GF677’ and ‘Viking’ from each block were harvested at each time point. Aboveground (leaf and stem) and root biomass of all hybrids were measured after all three harvests. Leaf and root morphological traits of all hybrids were measured at 30 and 60 days of treatments, respectively. Lamina Cl^–^ concentration and leaf gas exchange of all hybrids were measured at 60 days of treatments.

### Tissue morphology and lamina Cl^–^ concentration

At both 30 and 60 days of treatments, all leaf, stem and root tissues were harvested respectively, and key leaf and fine-root traits were measured. Total leaf area was determined by detaching all leaves and measuring leaf area using the LI-3000C Portable Area Meter. After removing the stem by cutting at the soil surface, the entire contents (basal stem plus roots and 2 liters of soil) were removed from the pot and then cut longitudinally from the middle. A radial segment representing approximately one eighth of the soil volume was taken as a subsample and stored in the dark at 3°C until processed. The roots were gently washed from the sub-sample using water and a fine mesh sieve (0.2 mm). There was no taproot for any plant sampled in this study. Root traits (root length, surface area, average diameter, and volume) were then determined using a scanning and digital image analysis system (WinRhizo; Régent Instruments, Quebec, Canada). Total root length was determined from the root length at each radial segment sampling divided by the ratio between the sampled root dry mass and the plant total root dry mass. In addition, root length was apportioned into 10 root diameter classes (0*–*0.075 mm, 0.075*–*0.13 mm, 0.13*–*0.2 mm, 0.2*–*0.3 mm, 0.3*–*0.4 mm, 0.4*–*0.5 mm, 0.5*–*0.75 mm, 0.75*–*1 mm, 1*–*2 mm, and more than 2 mm) – based on the pattern of root order distribution at the initial scanning and analysis using WinRhizo – to investigate the distribution of fine-root length according to fine-root diameter classes.

Following trait measurements, the leaf and root samples were oven-dried at 60°C for at least 48 h and weights were recorded after samples reached constant weight. The dried leaf tissue of all plants at 60 days of treatments was ground into a powder and analyzed for lamina Cl^–^ concentration (mg g^-1^ dw) as described by Li et al. (2015). Specific leaf area (SLA, cm^2^ g^-1^) was calculated as the ratio between fresh leaf area and leaf dry mass. Specific root surface area (SRA, m^2^ kg^-1^) was calculated as the ratio between root surface area and dry mass (for all roots < 2 mm diameter). Root tissue density (RTD, g cm^-3^) was calculated as the ratio between root dry mass and fresh volume (for all roots < 2 mm diameter). Percentage of root length within each diameter class relative to the total root length was also calculated.

### Stem water potential and leaf gas exchange

At 60 days of treatments, prior to the morphological trait determination, measurements of midday stem water potential (Ψ_stem_) and leaf gas exchange were also conducted. Mature leaves were enclosed in reflective, opaque, plastic bags (PMS Instrument Company, Corvallis, OR, USA) for one hour before the midday Ψ_stem_ measurements were determined using a pressure chamber (Soil moisture Corp, Santa Barbara, CA, USA). Ψ_stem_ measurement was not determined for ‘Nemaguard’ due to its small petiole size. Leaf gas exchange measurements were performed on the same day using young, fully expanded, sun-exposed leaves, with a portable photosynthesis system (LI-6400, Li-Cor Inc., Lincoln, NE, USA). Before each measurement, the leaf was acclimated in the chamber for 5 to 10 minutes to achieve stable gas exchange readings, with leaf temperature maintained at 25°C, reference CO_2_ concentration controlled at 400 µmol CO_2_ mol^–1^ air, and a saturating photosynthetic photon flux density (*Q*) of 1800 µmol photon m^–2^ s^–1^. Vapor pressure deficit (*D*) was held as constant as possible during the measurement (*D* = approximately 1.5 kPa). After the leaf acclimated to the cuvette environment, the light-saturated net CO_2_ assimilation rate (*A*_sat_) and stomatal conductance (*g*_s_) were recorded. The intrinsic water use efficiency (WUE_i_) was calculated as the ratio between *A*_sat_ and *g*_s_ to represent the instantaneous balance between photosynthesis and transpiration.

### Statistical analyses

All statistical analyses were conducted in R. Homogeneity test and normality test were conducted before the analysis of variance. The package lm() was used to fit linear models and the package anova() was used to compute analysis of variance for the fitted models to assess the effects of hybrid, treatment, treatment duration, and their interactions. The package HSD.test() was used to make multiple means comparisons by means of Tukey’s HSD test. Principal components analysis (PCA) was conducted on nine traits – lamina Cl^–^ concentration, SLA, SRA, RTD, average root diameter, percentage of root length within the finest diameter class, *A*_sat_, *g*_s_ and WUE_i_ – in six *Prunus* hybrids after 60 days of watering treatments to investigate the trait salinity-response relations.

## Results

### Effects on lamina Cl^–^ concentration, plant growth, and leaf physiology

**Figure 1:**
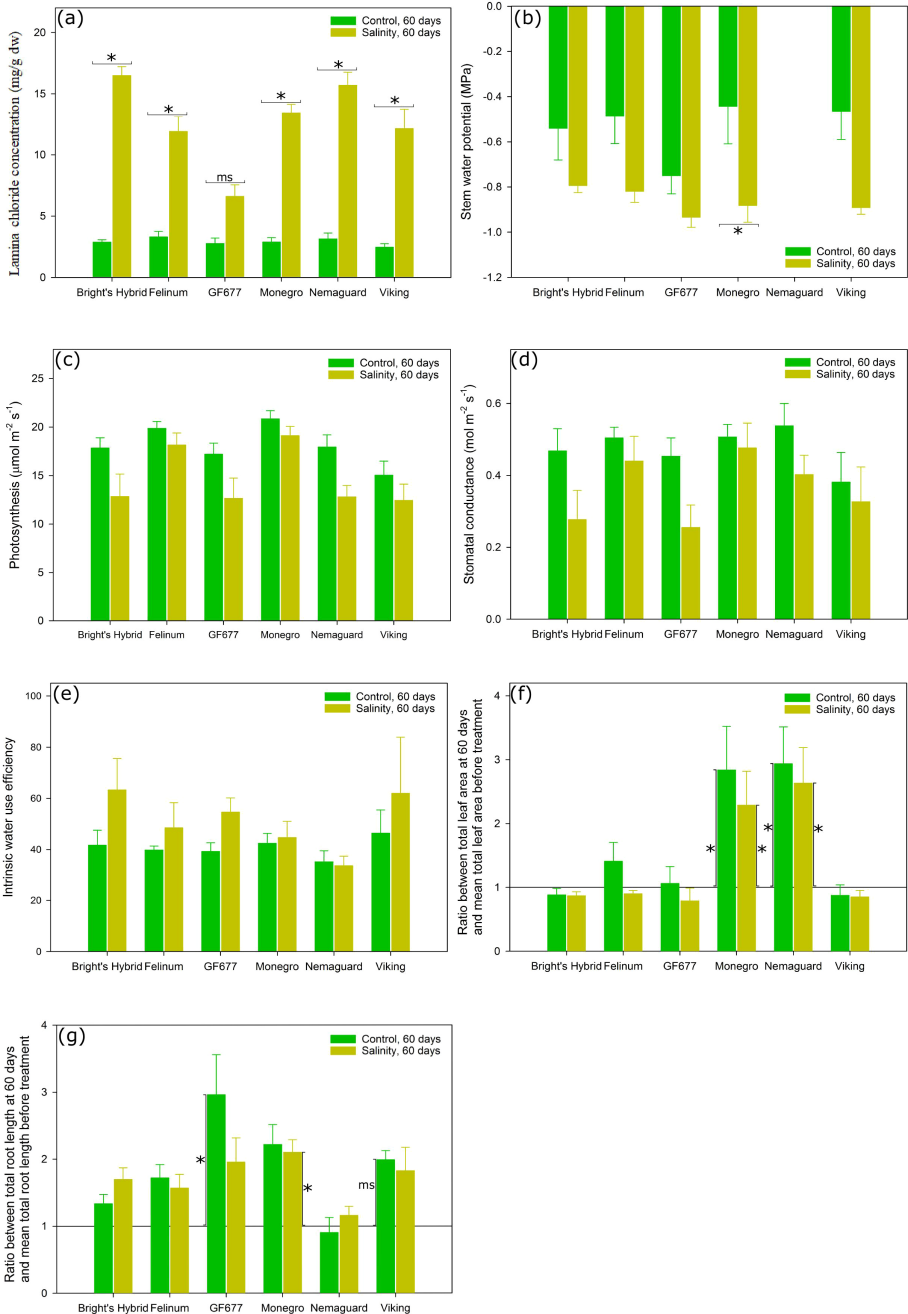
**(A)** Lamina Cl^–^ concentration, **(B)** stem water potential, **(C)** leaf net photosynthesis at saturating light (*A*_sat_), **(D)** stomatal conductance (*g*_s_), **(E)** intrinsic water use efficiency (WUE_i_), **(F)** the ratio between the plant total leaf area at 60 days and the mean total leaf area before treatment, and **(G)** the ratio between the plant total root length at 60 days and the mean total root length before treatment, for six *Prunus* hybrids exposed to control (reverse osmosis water) versus salinity treatment (3.3 dS m^-1^ Cl^–^ solution with mixed cations) for 60 days. The stem water potential measurement for ‘Nemaguard’ was not applicable due to small leaf size. The horizontal background line in **(F, G)** represents the ratio equal to 1. Values are means ± SE (*n* = 6 for ‘Bright’s Hybrid’, ‘Felinum’, ‘Monegro’ and ‘Nemaguard’; *n* = 5 for ‘GF677’ and ‘Viking’). Horizontal brackets and asterisks denote significant differences between two watering treatments for the same hybrid after 60 days. Vertical brackets and asterisks in subplot **(F, G)** denote significant differences between plants before and after 60 days of treatment for a given treatment. Significant differences in each case are indicated as ^ms^ *P* < 0.1 (marginal significance) or ^*^ *P* < 0.05.

There was a significant interaction between hybrid and treatment effects on lamina Cl^–^ concentration (**Table 1**). Compared with plants under control treatment for 60 days, plants of the six *Prunus* hybrids kept under salt treatment for the same duration consistently showed higher lamina Cl^–^ concentration (*P* < 0.1 for ‘GF677’, *P* < 0.05 for the other five hybrids) (**Figure 1A**) but unchanged *A*_sat_, *g*_s_, or WUE_i_ (**Figure 1C-E**, respectively). Salt-treated ‘GF677’ plants had loaded significantly less Cl^–^ in leaves compared to salt-treated plants of the other five hybrids after 60 days. After 60 days of salt treatment, only ‘Monegro’ showed more negative Ψ_stem_ relative to that for plants under control treatment (**Figure 1B**; measurement not applicable on ‘Nemaguard’).

**Table 1:** *P*-values of Tukey’s HSD test of comparison across six *Prunus* hybrids exposed to two watering treatments (reverse osmosis water versus 3.3 dS m^-1^ solution) for 30 days and 60 days of treatments.

	Lamina Cl^–^ concentration	Ψ_stem_	*A*_sat_	*g*_s_	WUE_i_	SLA	SRA	RTD	Average root diameter	Percentage of root length within 0.075–0.13 mm
Hybrid	**<0.001**	0.135	**<0.001**	0.090	0.242	**<0.001**	**0.040**	**<0.001**	**<0.001**	**<0.001**
Treatment	**<0.001**	**<0.001**	**<0.001**	**0.004**	**0.047**	**0.002**	0.370	0.369	0.495	0.972
Time	NA	NA	NA	NA	NA	**<0.001**	**0.008**	0.341	**<0.001**	**<0.001**
Hybrid × Treatment	**<0.001**	0.368	0.622	0.677	0.740	0.340	0.273	**0.044**	0.22	0.241
Hybrid × Time	NA	NA	NA	NA	NA	0.193	**0.006**	**0.001**	**<0.001**	**<0.001**
Treatment × Time	NA	NA	NA	NA	NA	**0.014**	0.671	0.067	**0.015**	**0.032**
Hybrid × Treatment × Time	NA	NA	NA	NA	NA	0.182	0.209	0.467	**0.035**	0.690

The traits include: lamina Cl^–^ concentration, Ψ_stem_, stem water potential; *A*_sat_, leaf net photosynthesis at saturating light; *g*_s_, stomatal conductance; WUE_i_, intrinsic water use efficiency; SLA, specific leaf area; SRA, specific root surface area; RTD, root tissue density; average root diameter, and the fraction of root length within the finest root diameter class (0.075–0.13 mm).

**Figure 2:**
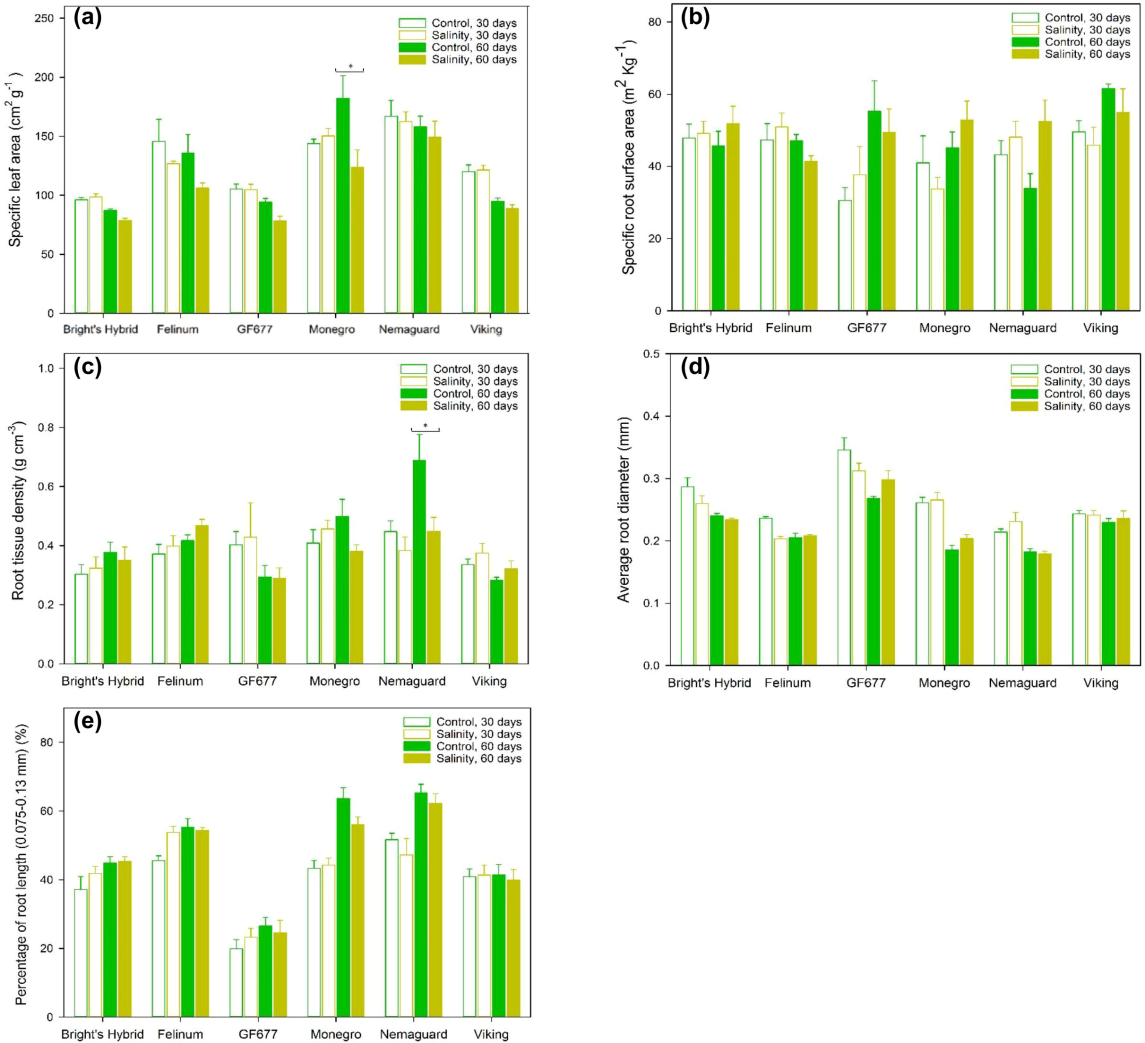
**(A)** Specific leaf area (SLA), **(B)** specific root surface area (SRA), **(C)** root tissue density (RTD), **(D)** average root diameter and **(E)** percentage of root length within the finest diameter class (0.075*–*0.13 mm) for six *Prunus* hybrids exposed to control (reverse osmosis water) versus salinity treatment (3.3 dS m^-1^ Cl^–^ solution with mixed cations) for 60 days. Values are means ± SE (*n* = 6 for ‘Bright’s Hybrid’, ‘Felinum’, ‘Monegro’ and ‘Nemaguard’; *n* = 5 for ‘GF677’ and ‘Viking’). Brackets and asterisks denote significant differences between two watering treatments for the same hybrid after 60 days, indicated as ^*^ *P* < 0.05.

**Figure 3:**
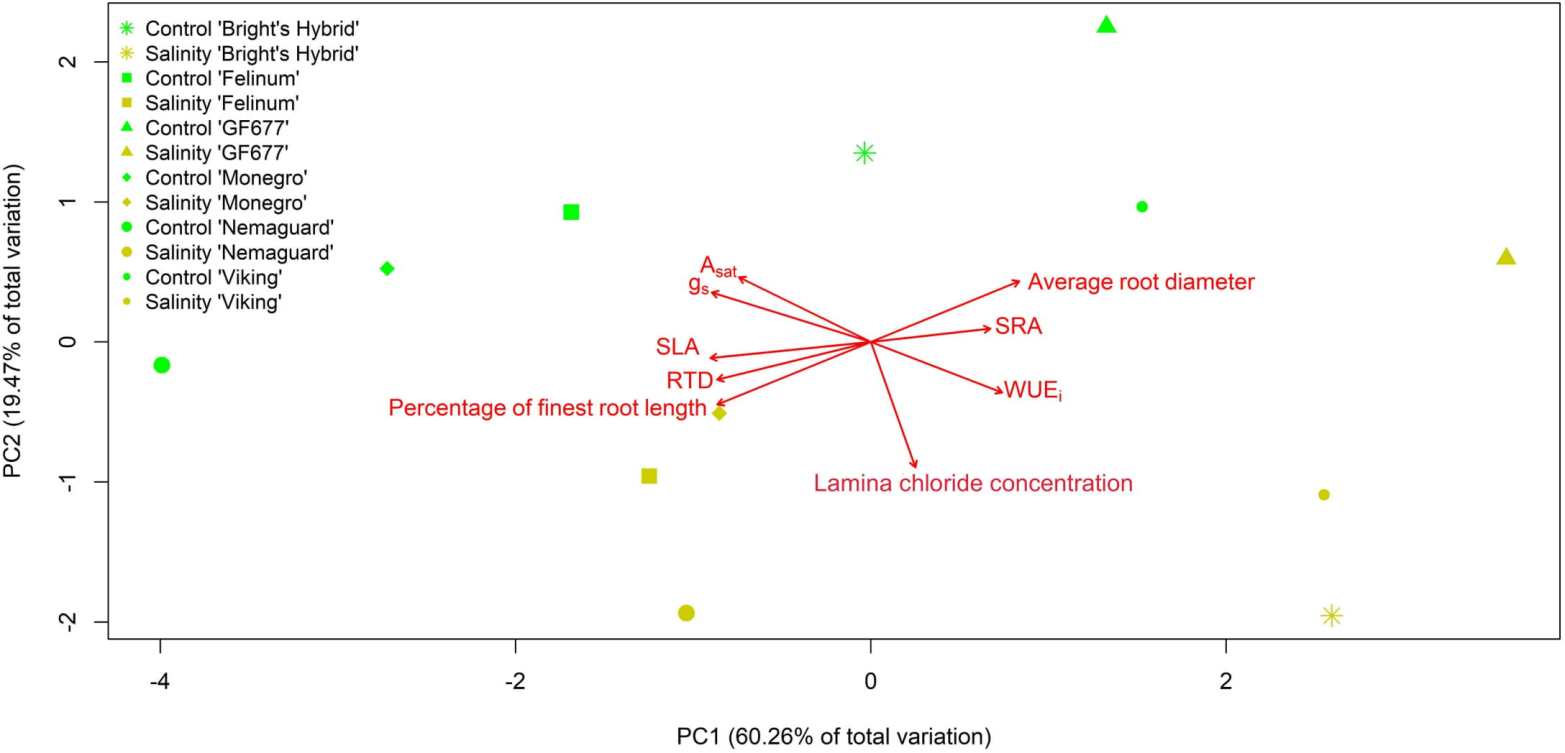
Differential salt-stress responses of leaf and fine-root traits depicted by principal components analysis (PCA) conducted on nine traits of six *Prunus* hybrids exposed to control (reverse osmosis water) versus salinity treatment (3.3 dS m^-1^ Cl^–^ solution with mixed cations) for 60 days. The traits are lamina Cl^–^ concentration, leaf net photosynthesis at saturating light (*A*_sat_), stomatal conductance (*g*_s_), intrinsic water use efficiency (WUE_i_), specific leaf area (SLA), specific root surface area (SRA), root tissue density (RTD), average root diameter, and the percentage of root length within the finest diameter class (0.075*–*0.13 mm). The first principal component (PC1) explained 60.26% of the total variation, and the second principal component (PC2) explained 19.47% of the total variation.

Data were, respectively, shown in **Figures 1**–**3**; **Supplementary Figures S2**-**S4**. NA is not applicable because data were not able to be collected at 30 days due to resource limitations. Bold values indicate significant effect where *P* < 0.05.

The linear regression analysis on aboveground biomass, root biomass and whole plant biomass along the three harvest time points showed that the root biomass of salt-treated ‘Nemaguard” plants was the only one exhibiting a negative relationship with the duration of treatment (**Supplementary Figure S1A–C**). Compared with plants before treatments, none of the six *Prunus* hybrids showed a significant change of total leaf area or total root length after 30 days of treatment (**Supplementary Figures S2**, **S3**). However, after 60 days, ‘Monegro’ and ‘Nemaguard’ plants – in both control and salinity treatments – had significantly increased their total leaf area compared with plants before treatment (**Figure 1F**). The prolonged salt treatment – but not control treatment – also led to a significant increase of total root length in ‘Monegro’ plants compared with plants before treatment (**Figure 1G**). The prolonged salt treatment led to an increase of total root length in ‘GF677’ (*P* < 0.05) and ‘Viking’ (*P* < 0.1) plants compared with plants before treatment (**Figure 1G**).

### Effects on SLA and SRA

Compared to plants under control treatment, no hybrid under salt treatment showed significantly modified SLA or SRA after 30 days (**Figures 2A, B**; **Supplementary Figure S4**). After 60 days of salt treatment, ‘Monegro’ plants showed a significant reduction of SLA, while the other five hybrids did not modify their SLA (**Figure 2**). No hybrid showed significantly modified SRA after 30 or 60 days of salt treatment (**Figures 2A, B**; **Supplementary Figure S4**). ‘Nemaguard’ plants showed an increase of SRA after 60 days of salt treatment (not significant; **Figure 2B**).

### Effects on RTD, average root diameter and root diameter distribution

There was a significant interaction between hybrid and treatment effects on RTD (**Table 1**). There was a significant interaction between hybrid and treatment and time effects on average root diameter (**Table 1**). After 30 days of salt treatment, none of the six hybrids showed a significant change in RTD (**Figure 2C**). However, after 60 days of salt treatment, ‘Nemaguard’ showed significantly lower RTD relative to plants under control treatment (**Figure 2C**). Compared to plants under control treatment, none of the six hybrids under salt treatment showed a significant change of average root diameter (**Figure 2D**) or the percentage of root length within finest diameter class after 30 or 60 days (**Figure 2E**; **Supplementary Figure S4**).

Salt-treated ‘GF677’ had significantly higher average root diameter compared to salt-treated plants of the other five hybrids at both 30 days (*P* < 0.1 when compared with ‘Monegro’, *P* < 0.05 when compared with the other four hybrids) and 60 days (*P* < 0.05) (**Figure 2D**). Five of the six hybrids showed higher fraction of root length within the finest diameter class (0.075*–*0.13 mm), except ‘GF677’ whose majority of total root length was within the diameter classes 0.13*–*0.2 mm (**Figure 2E**; **Supplementary Figure S4**). No hybrid under salt treatment showed concurrent significant modifications of the fine-root traits depicting root fineness – SRA (**Figure 2B**), average root diameter (**Figure 2D**) and root diameter distribution (**Figure 2E**; **Supplementary Figure S4**).

### Response interrelations among leaf and fine-root traits, leaf gas exchange and lamina Cl^–^ concentration

PCA was dominated by the first principal component (PC1), which explained 60.26% of the total variation (**Figure 3**). PC1 was characterized by a hybrid-dependent modification of SLA, SRA, RTD, average root diameter and percentage of root length within the finest diameter class. The salinity responses of SRA and average root diameter were negatively correlated with the SLA, RTD and the percentage of root length within the finest diameter class.

The second principal component (PC2) explained 19.47% of the total variation. PC2 tended to be largely driven by increased lamina Cl^–^ concentration in salt-treated plants of all six hybrids. PC2 showed a gradient from control to salt-treated plants for all six hybrids after 60 days of treatments, characterized by the positive correlation between lamina Cl^–^ concentration and WUE_i_, and by the negative correlation between lamina Cl^–^ concentration and *A*_sat_ and *g*_s_ (**Figures 1**, **3**). Leaf gas exchange variables tended to contribute to both PC1 and PC2.

PCA showed that the six hybrids responded similarly in terms of increasing lamina Cl^–^ concentration and decreasing leaf gas exchange, and differentially in terms of modifying leaf and/or root traits (**Figure 3**). Control plants of all six hybrids were aligned to the left-top part, while salt-treated plants of all six hybrids were aligned to right-bottom part of **Figure 3** *–* a pattern tended to be driven by the interaction between hybrid and treatment. The *a priori* known gradient of salinity-sensitivity among hybrids was reflected more along PC1 (**Figure 3**), with the most sensitive hybrid ‘Nemaguard’ aligning on the left side of PC1, and the three salinity-tolerant hybrids – ‘Bright’s Hybrid’ (El-Motaium et al., 1994), ‘Viking’ (Company and Gradziel, 2017) and ‘GF677’ (Najafian et al., 2008; Ouraei et al., 2009; Dejampour et al., 2012) aligning on the right side of PC1 (**Figure 3**).

## Discussion

This study displays important empirical evidence that (1) prolonged but not short-term salt treatment can lead to the hybrid-dependent modification of leaf and fine-root acquisitive traits, decoupling from lamina Cl^–^ concentration (since all hybrids accumulated Cl^–^ in laminae during salt treatment, albeit to different levels), and (2) congeneric hybrids can show different above- and below-ground trait response combinations under prolonged salt treatment.

### Chloride accumulation in reflecting the hybrid-specific salinity tolerance

Shoot Cl^–^ exclusion is thought to be a constitutive rather than an inducible trait, with rootstock effects sometimes seen at low salinity (control) treatments (Zhou-Tsang et al., 2021). Laminae Cl^–^ concentration of the control plants in this study was not significantly different among the hybrids (**Figure 1A**). On the other hand, compared to control plants, there were significantly higher lamina Cl^–^ concentration in salt-treated plants of all six hybrids (**Figure 1A**), supporting previous studies on ion accumulation in response to salt treatment (e.g. Romero-Aranda et al., 2001; Sandhu et al., 2020; Lupo et al., 2022).

When comparing the hybrid-specific lamina Cl^–^ concentration, salt-treated ‘GF677’ loaded relatively lower concentration than the other five salt-treated hybrids (**Figure 1A**), underlying the significant hybrid and treatment interaction effect on lamina Cl^–^ concentration (**Table 1**). This may be evidence of superior root-based capacity of ‘GF677’ for Cl^–^ exclusion compared to the other five hybrids. Ideally, the Cl− exclusion capacity of hybrids is best compared by undertaking a complete analysis of the concentrations of Cl^–^ (and similarly for other ions such as Na^+^) in the whole-plant and composite organs (e.g., lamina, petiole, stem and root) (Walker et al., 2024), however, such detailed analysis was not possible in this study.

The six hybrids used in the study have different genetic backgrounds which likely underlie different capacities to regulate the accumulation of Cl^–^. Various studies have attempted to understand the molecular basis of Cl^–^ accumulation in plants (Wu and Li, 2019). There is evidence for both single gene and multi-gene (Gong et al., 2011; Fort et al., 2015) control of Cl^–^ exclusion and various candidate genes have been identified (Wu and Li, 2019).

### Fine-root and leaf acquisitive traits in reflecting the hybrid-specific salinity tolerance

This study demonstrates important hybrid-specific below-ground strategies after prolonged salt treatment. Sixty (60) days but not 30 days of salt treatment led to diverse trade-offs among fine-root traits (SRA, RTD, average root diameter and the fine-root distribution; **Figures 2**, **3**). Plant root system is plastic in adaptation to saline soil (Lupo et al., 2022; Shelden and Munns, 2023). Plants can utilize diverse combinations of modifications of fine-root traits such as SRA, RTD and/or average root diameter to maximize root resource acquisition in adaptation to environmental stress (Ostonen et al., 2007; Valverde-Barrantes and Blackwood, 2016; Zhou et al., 2020; Lupo et al., 2022). For instance, plants can construct fine-roots of high SRA deriving independently from lower RTD and/or thinner root diameter under soil drought (Kramer-Walter et al., 2016) and salinity conditions (Lupo et al., 2022).

In this study, salt-treated plants of the *a priori* known most-sensitive hybrid ‘Nemaguard’ not only showed a negative relationship between root biomass and salt treatment duration but also showed decreased RTD while the other five salt-treated hybrids did not (**Figure 2C**). The latter underlies the significant hybrid and treatment interaction effect on RTD (**Table 1**). The decreased RTD in the salt-treated ‘Nemaguard’ – whose average root diameter (and diameter distribution) remained unchanged – relative to that for plants under control treatment (*P* < 0.05; **Figure 2**), was linked with an increase of mean value of SRA (though not significant; **Figure 2**). SRA is a proxy of plant resource absorptive capacity, with various plant species shown to be capable of developing root systems with increased SRA to support greater nutrient acquisition under environmental stress (Aerts and Chapin, 1999; Lupo et al., 2022; Zhao et al., 2024). The root system of salt-treated ‘Nemaguard’ with higher SRA was theoretically less expensive for plants to construct and maintain, in terms of carbon investment per unit of root surface area for soil resource foraging and acquisition, as a trade-off with decreased lifespan reflected by decreased RTD (Zhou et al., 2020; Zhao et al., 2024).

These results support previous studies reporting hybrid-specific increase of SRA under salinity treatment (Rewald et al., 2011; Lupo et al., 2022). Salt-stressed plants can shift the resource investment strategy towards a resource-safer mode through investing relatively more resource into new tissue (e.g., new roots) to explore new space for acquiring resource (Robin et al., 2016). In this study, salt-treated ‘Nemaguard’ plants tended to build fine-root with higher SRA when the photosynthesis was notably reduced by salt treatment.

This study also provides evidence supporting hybrid-specific salinity-response coordination among leaf traits. When compared with plants under control treatment, salt-treated ‘Monegro’ plants with lower Ψ_stem_ maintained photosynthesis and total leaf area but decreased SLA – the leaf area built per unit of leaf mass invested (**Figures 1**, **2**). The effect of decreased leaf water potential on leaf growth can be independent from that on photosynthesis (Boyer, 1970; Tardieu et al., 2011), as there are factors other than photosynthate availability limiting leaf growth (Boyer, 1970). Plants with a decreased water potential could develop smaller leaves (Romero-Aranda et al., 2001) or maintain the leaf area unchanged (Cavender-Bares, 2019; Rowland et al., 2023).

### Interconnection between responses of lamina chloride concentration and traits

This study also provides evidence that the intraspecific differential Cl^–^ loading could be related to intraspecific differential root diameter distribution. Roots of smaller diameter could have greater ion uptake capacity (Eissenstat, 1992) and function better in foraging and absorption (Fitter, 1994). Salt-treated ‘GF677’ had higher average root diameter compared to salt-treated plants of the other five hybrids at both 30 days and 60 days (**Figure 2**). Results on root diameter distribution showed that the highest fraction of root length in ‘GF677’ fell in the diameter class 0.13–0.2 mm, while the other five hybrids showed their highest fraction of root length within the finest diameter class (0.075*–*0.13 mm) (**Supplementary Figure S3**).

The positive correlation between lamina Cl^–^ concentration and WUE_i_ in **Figure 3** can be compared with previous observations of a link between genotype capacity for Cl^–^ exclusion and water use efficiency. For example, higher yield (Walker et al., 2002) and higher water use efficiency (Walker et al., 2005) were recorded by a better Cl^–^ excluding rootstock at higher salinity (3.8 dS m^-1^) compared to a poorer Cl^–^ excluding genotype (i.e., ‘Sultana’ grafted on the efficient Cl^–^ excluder ‘Ramsey’ rootstock compared to the inefficient excluder ‘Sultana’ on own roots).

The results for leaf and fine-root traits provide important evidence of interspecific differential resource investment strategies in modifying above- and/or below-ground traits to help plants cope with salt stress, which helps disentangle the mixed evidence showing that below-ground organs are more salinity-sensitive than above-ground organs (e.g., Bernstein et al., 2004), or vice versa (e.g., Munns and Tester, 2008). The hybrid-dependent variation in salinity-induced plasticity of SLA and fine-root traits after 60 days, reflects hybrid-specific combinations of above- and/or below-ground trait-based responses for plants to adapt to salinity treatment. The gradient of salinity responses of lamina Cl^–^ concentration along PC2 in **Figure 3** was mainly driven by the treatment effect, while the gradient along PC1 tended to be driven more strongly by the hybrid effect (**Figure 3**). No salt-treated hybrid showed concurrent significant modifications of both SLA and SRA after 60 days, against the expectation that stress responses of SLA and SRA would be analogous to each other (Eissenstat et al., 2000). This may be because root traits are shaped by belowground environmental constraints which are different from aboveground constraints shaping leaf traits (Zhao et al., 2024).

Despite a lack of significant interaction effects between hybrid and treatment on a single trait (**Table 1**, **Figures 1**, **2**), this study highlights a clear intra-specific order of all traits in response to salt stress (**Figure 3**). These results support previous studies (e.g., Robin et al., 2016; Lupo et al., 2022) suggesting that crop varieties cannot be classified as salinity tolerant or intolerant merely according to performance of a trait. The salinity response of different traits can be variety-dependent, associated with different genes governing the expression of these traits with trait-specific salt-stress tolerance or vulnerability (Robin et al., 2016). Besides, the decoupled relationship between trait-response and Cl^–^ accumulation, highlights that the differential intraspecific salinity tolerance cannot be reflected by solely considering tissue ion accumulation. The decoupling between the salinity responses of lamina Cl^–^ concentration and plant acquisitive traits could be associated with the extent to which the hybrid and treatment interact, underlying the evidence that the extent to which plant acquisitive traits respond to treatment is largely affected by hybrid.

These results suggested that both the salt effects on plant growth and acquisitive traits can be used to evaluate the salinity tolerance of *Prunus* hybrids. In this paper, the six hybrids were compared for their differential Cl^-^ exclusion and for differential salt tolerance. The laminae Cl^-^ data suggested that ‘GF677’ was a relatively better Cl^-^ excluder than the other five hybrids under salt treatment. On the other hand, for potted plant studies, growth and physiological traits under salt treatment are the primary traits that can be used to compare salt tolerance of genotypes. **Supplementary Figure S1** showed a negative root-biomass relationship to salt treatment for ‘Nemaguard’ relative to that of the other five hybrids. The study further examined whether there were any links between the salt-response of plant biomass and that of fine-root and leaf acquisitive traits, which highlighted that salt-treated ‘Nemaguard’ modified their fine-root acquisitive traits. These results suggested that the effects of salinity treatment duration on both root biomass and fine-root traits may reflect the apparent greater salt sensitivity of ‘Nemaguard’ (El-Motaium et al., 1994; Company and Gradziel, 2017)compared to the other five hybrids.

This pot study under controlled conditions provides meaningful insights on the salinity-response interrelationships among lamina Cl^–^ concentration and leaf and fine-root acquisitive traits at the intraspecific level. The study contributes to better understanding of salinity effects on tree crop saplings, which can be particularly useful for rootstock-dependent salinity-sensitive fruit crops (e.g., almond, avocado, grapevine), whose salinity management has been identified as a priority industry challenge (Bernstein et al., 2004; Newett et al., 2022; Lupo et al., 2022). For instance, the performance of almond plantings under salt stress is largely determined by *Prunus* rootstock hybrids with differential salinity tolerance (Company and Gradziel, 2017), as the degree of salinity tolerance of the rootstock is critical for the performance of the scion grafted with the rootstock (Gainza et al., 2015; Lupo et al., 2022). The findings of this pot-based study remain to be validated in field conditions, incorporating considerations of the key production factors such as climate, growth stage, soil volume, soil type, drainage, and so on.

## Data Availability

The raw data supporting the conclusions of this article will be made available by the authors, upon reasonable request.

## Supplementary material

Supplementary Figure 1 Aboveground biomass **(A)**, root biomass **(B)** and whole plant biomass **(C)** at three harvest time points (D0: the day before harvest; D30: 30 days of treatment; D60: 60 days of treatment) for six *Prunus* hybrids (Bright’s Hybrid’: BH; ‘Felinum’: FE; ‘GF677’: GF; ‘Monegro’: MO; ‘Nemaguard’: NE; ‘Viking’: VI) exposed to control treatment (reverse osmosis water) or salinity treatment (3.3 dS m^-1^ Cl^–^ solution with mixed cations). Dots represent raw data. Above the bar plots, brackets and asterisks denote significant differences among three harvest time points for the same hybrid under the same treatment, indicated as **P* < 0.05 and ***P* < 0.01. Linear regression analysis outputs were shown below the bar plots. *R* ^2^ represents the coefficient of determination, which determines the strength of the linear model besides the direction of the correlation.

Supplementary Figure 2 The ratio between the plant total leaf area after 30 days of treatments (reverse osmosis water versus 3.3 dS m^-1^ Cl^–^ solution with mixed cations) and the mean total leaf area before treatment (horizontal background line represents a ratio equal to 1). Values are means ± SE (*n* = 6 for ‘Bright’s Hybrid’, ‘Felinum’, ‘Monegro’ and ‘Nemaguard’; *n* = 5 for ‘GF677’ and ‘Viking’).

Supplementary Figure 3 The ratio between the plant total root length after 30 days of treatments (reverse osmosis water versus 3.3 dS m^-1^ Cl^–^ solution with mixed cations) and the mean total root length before treatment (horizontal background line represents a ratio equal to 1). Values are means ± SE (*n* = 6 for ‘Bright’s Hybrid’, ‘Felinum’, ‘Monegro’ and ‘Nemaguard’; *n* = 5 for ‘GF677’ and ‘Viking’).

Supplementary Figure 4 Percentage of root length within 10 root diameter classes (0*–*0.075 mm, 0.075*–*0.13 mm, 0.13*–*0.2 mm, 0.2*–*0.3 mm, 0.3*–*0.4 mm, 0.4*–*0.5 mm, 0.5*–*0.75 mm, 0.75*–*1 mm, 1*–*2 mm, and more than 2 mm) at 60 days of treatments (reverse osmosis water versus 3.3 dS m^-1^ Cl^–^ solution with mixed cations). Values are means (*n* = 6 for ‘Bright’s Hybrid’, ‘Felinum’, ‘Monegro’ and ‘Nemaguard’; *n* = 5 for ‘GF677’ and ‘Viking’).
