# Supplementary figures and images for: Fine-root and leaf acquisitive traits decoupled from chloride accumulation in reflecting the differential salinity tolerance among Prunus hybrids
Source: Front Plant Sci. 2025 Jan 8;15:1502201. doi: 10.3389/fpls.2024.1502201 (PMC11750577; doi:10.3389/fpls.2024.1502201)

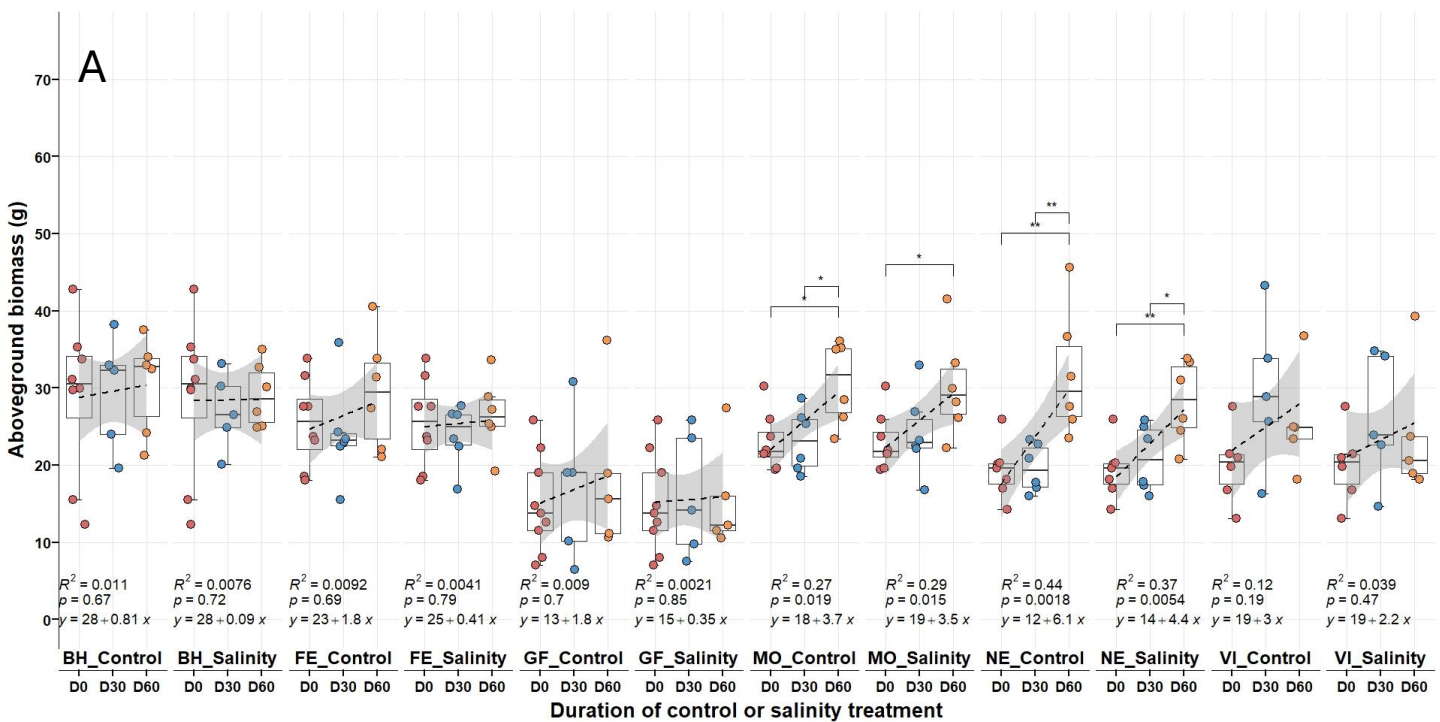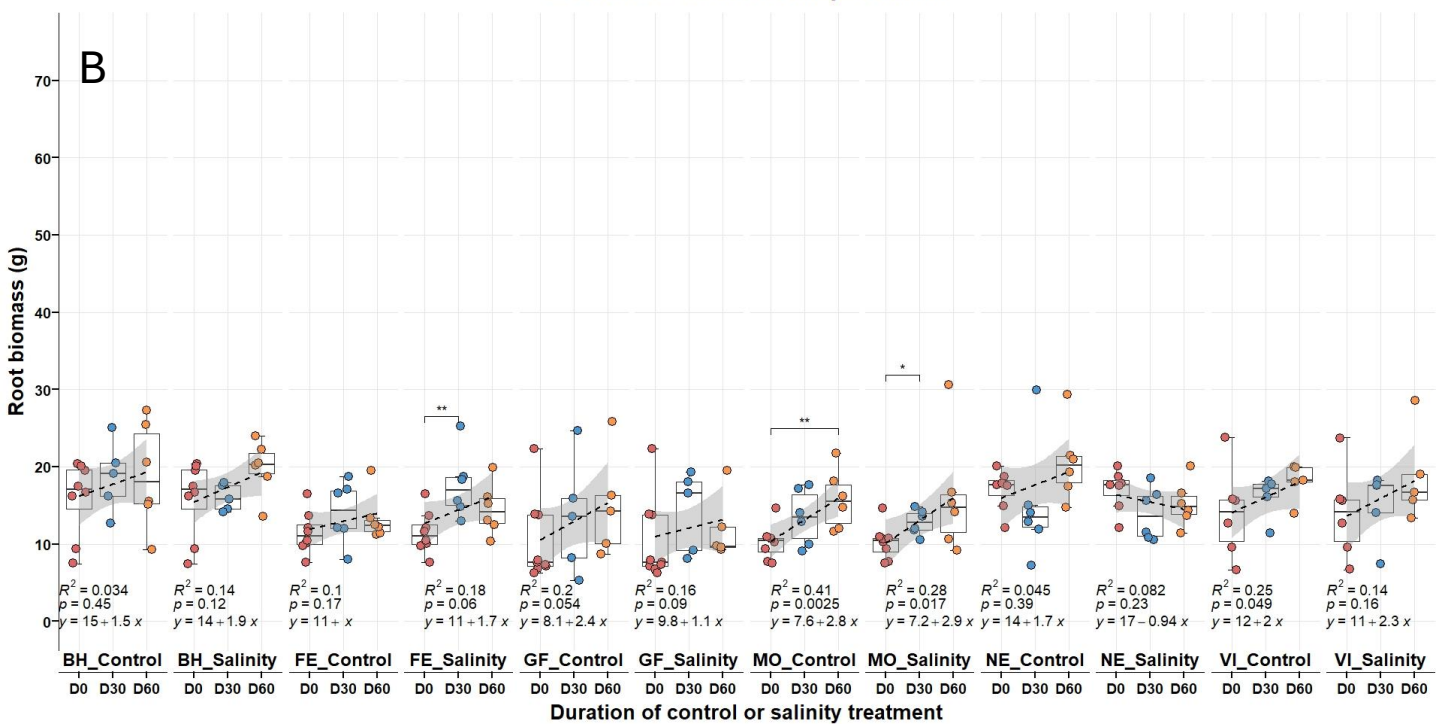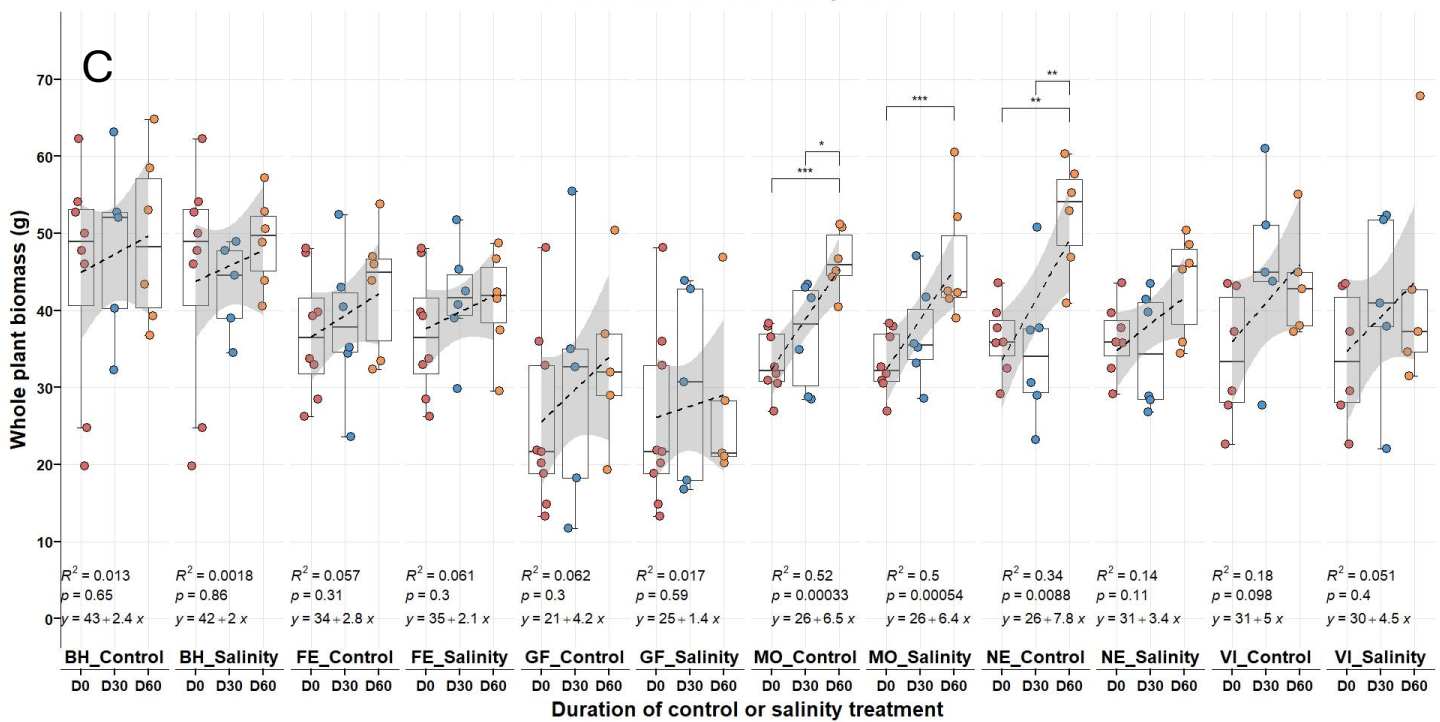

Supplement: Supplementary Figure 1 — Aboveground biomass (A), root biomass (B) and whole plant biomass (C) at three harvest time points (D0: the day before harvest; D30: 30 days of treatment; D60: 60 days of treatment) for six Prunus hybrids (Bright’s Hybrid’: BH; ‘Felinum’: FE; ‘GF677’: GF; ‘Monegro’: MO; ‘Nemaguard’: NE; ‘Viking’: VI) exposed to control treatment (reverse osmosis water) or salinity treatment (3.3 dS m-1 Cl– solution with mixed cations). Dots represent raw data. Above the bar plots, brackets and asterisks denote significant differences among three harvest time points for the same hybrid under the same treatment, indicated as *P < 0.05 and **P < 0.01. Linear regression analysis outputs were shown below the bar plots. R 2 represents the coefficient of determination, which determines the strength of the linear model besides the direction of the correlation. [file Image1.pdf]

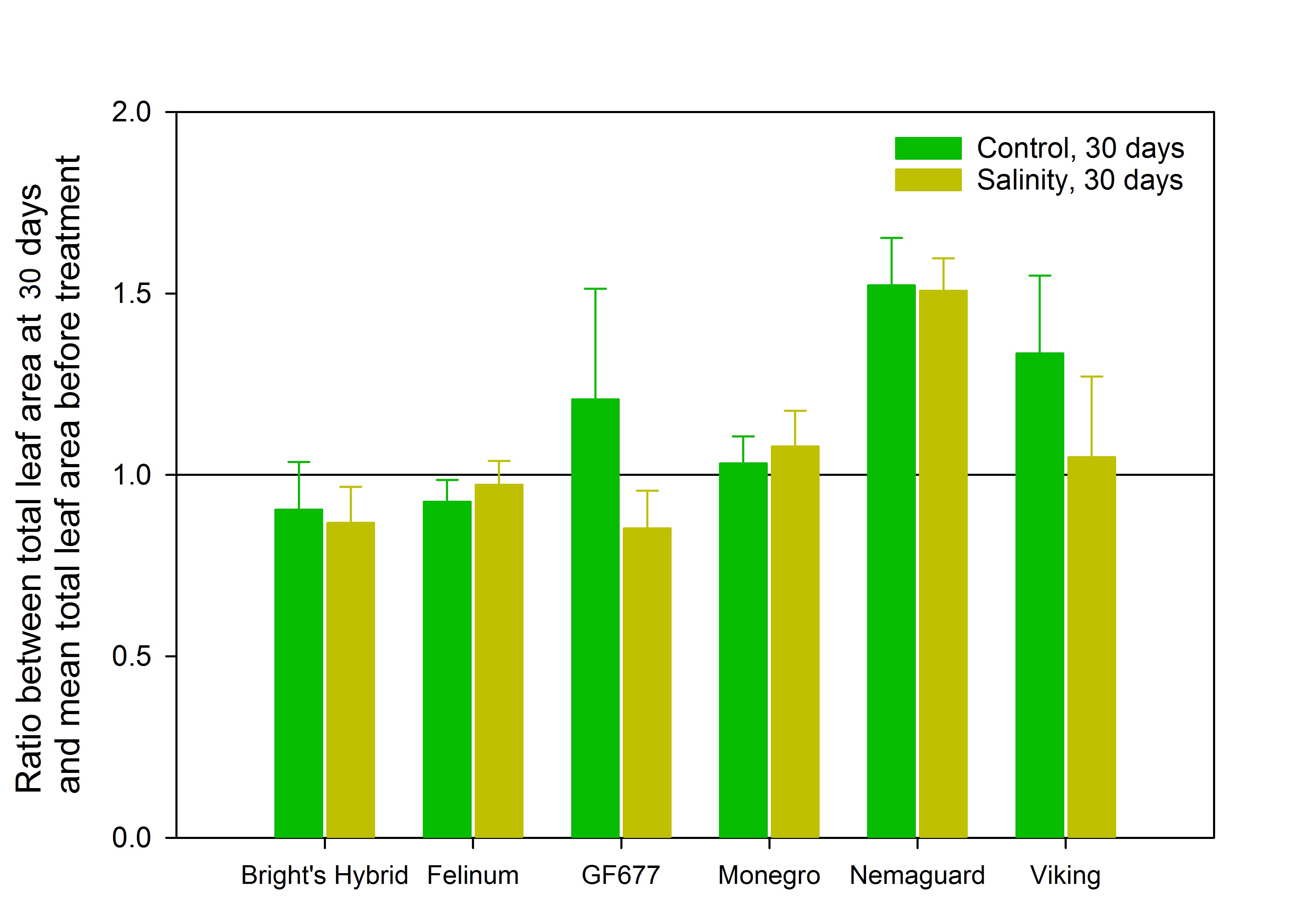

Supplement: Supplementary Figure 2 — The ratio between the plant total leaf area after 30 days of treatments (reverse osmosis water versus 3.3 dS m-1 Cl– solution with mixed cations) and the mean total leaf area before treatment (horizontal background line represents a ratio equal to 1). Values are means ± SE (n = 6 for ‘Bright’s Hybrid’, ‘Felinum’, ‘Monegro’ and ‘Nemaguard’; n = 5 for ‘GF677’ and ‘Viking’). [file Image2.jpeg]

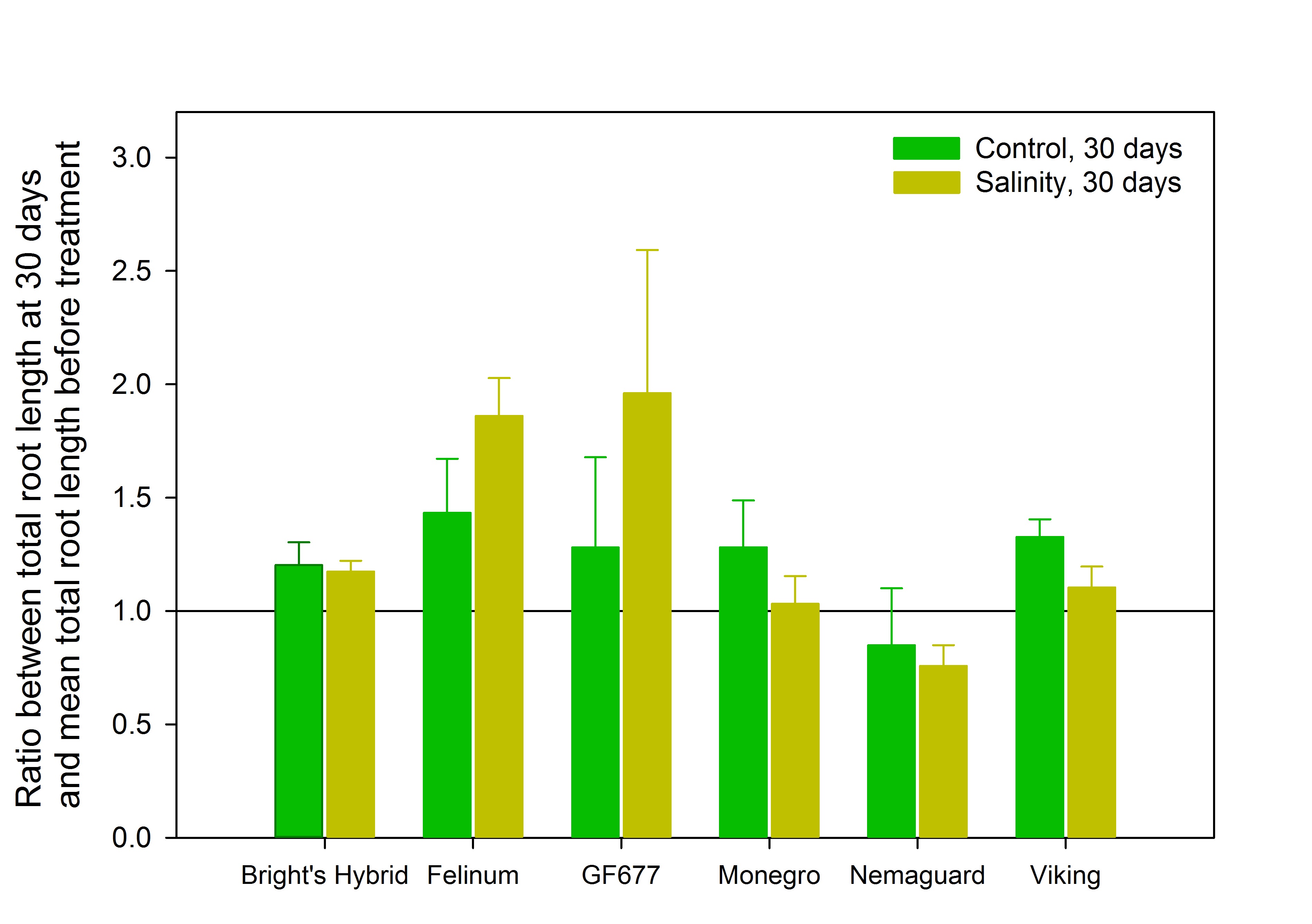

Supplement: Supplementary Figure 3 — The ratio between the plant total root length after 30 days of treatments (reverse osmosis water versus 3.3 dS m-1 Cl– solution with mixed cations) and the mean total root length before treatment (horizontal background line represents a ratio equal to 1). Values are means ± SE (n = 6 for ‘Bright’s Hybrid’, ‘Felinum’, ‘Monegro’ and ‘Nemaguard’; n = 5 for ‘GF677’ and ‘Viking’). [file Image3.jpeg]

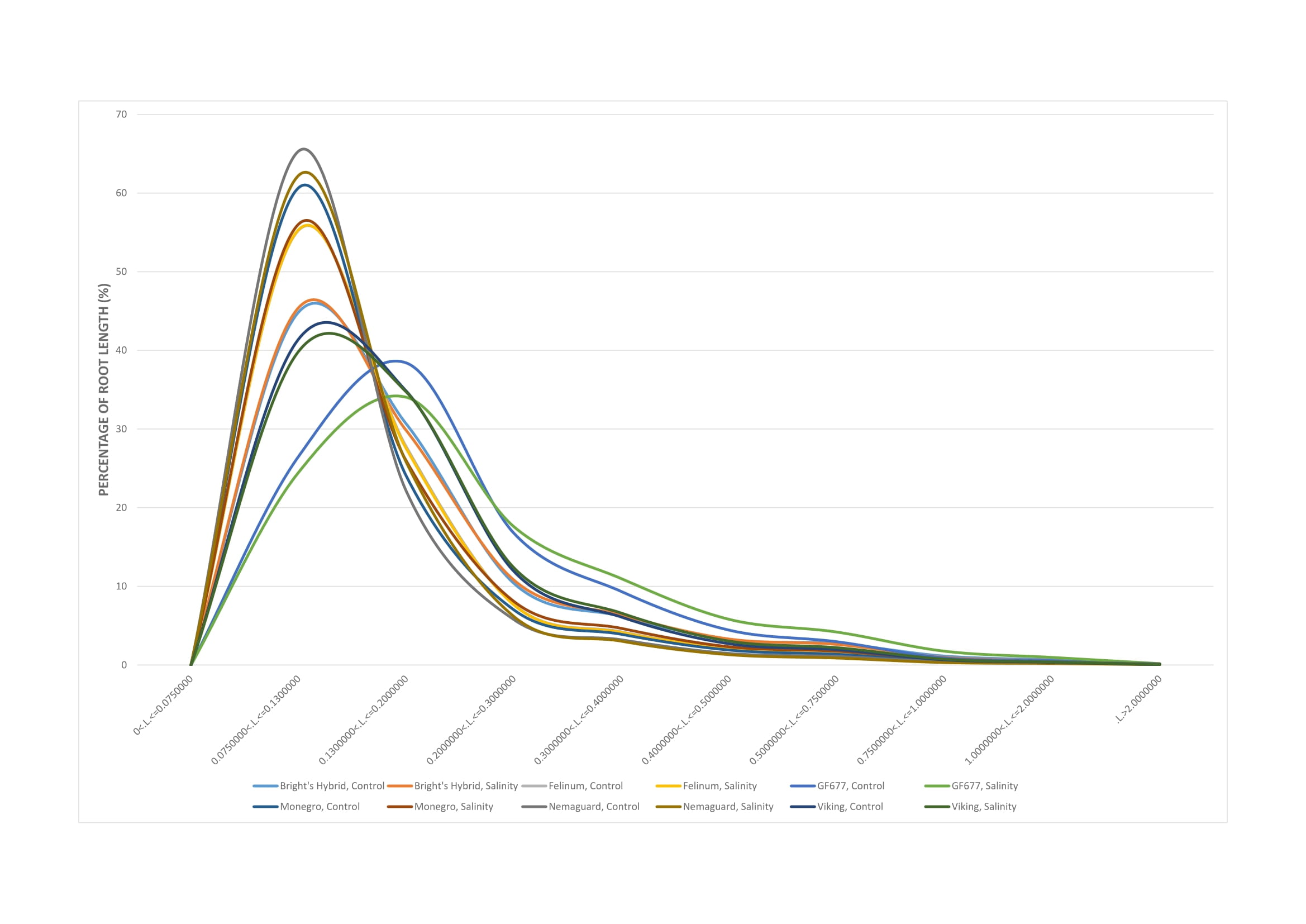

Supplement: Supplementary Figure 4 — Percentage of root length within 10 root diameter classes (0–0.075 mm, 0.075–0.13 mm, 0.13–0.2 mm, 0.2–0.3 mm, 0.3–0.4 mm, 0.4–0.5 mm, 0.5–0.75 mm, 0.75–1 mm, 1–2 mm, and more than 2 mm) at 60 days of treatments (reverse osmosis water versus 3.3 dS m-1 Cl– solution with mixed cations). Values are means (n = 6 for ‘Bright’s Hybrid’, ‘Felinum’, ‘Monegro’ and ‘Nemaguard’; n = 5 for ‘GF677’ and ‘Viking’). [file Image4.jpeg]
